# Intestinal inflammation in TNBS sensitized rats as a model of chronic inflammatory bowel disease

**DOI:** 10.1155/S0962935192000206

**Published:** 1992

**Authors:** N. Selve, T. Wöhrmann

**Affiliations:** 1 Department of Pharmacology Research Centre Grünenthal GmbH Zieglerstr. 6 Aachen W-5100 Germany; 2 Department of Toxicology and Pathology Research Centre Grünenthal GmbH Zieglerstr. 6 Aachen W-5100 Germany

## Abstract

An enteritis, based on a delayed-type hypersensitivity reaction, was induced in TNBS (2,4,4-trinitrobenzenesulphonic acid) sensitized rats by multiple intrajejunal challenge with TNBS via an implanted catheter. This treatment induced chronic inflammation of the distal small intestine characterized by intense hyperaemia, oedema and gut wall thickening as assessed by macroscopic scoring and weighing a defined part of the dissected intestine. Histologically, the inflammatory response included mucosal and submucosal cell infiltration by lymphocytes and histiocytes, transmural granulomatous inflammation with multinucleated cells and activated mesenteric lymph nodes. Ex vivo stimulated release of the inflammatory mediator LTB_4_ in the dissected part of the intestine was increased following TNBS treatment. Drug treatment with sulphasalazine or 5-aminosalicylic acid improved the enteritis score and attenuated TNBS induced oedema formation and LTB_4_ production. The applicability and relevance of this new model are discussed with respect to drug development and basic research of inflammatory bowel diseases.


Mediators of Inflammation, 1, 121–126 (1992)

### Intestinal inflammation in TNBS sensitized rats as a model of chronic inflammatory bowel disease

N. Selve[1,CA] and T. Wöhrmann[2]

[1]Department of Pharmacology and [2]Department of Toxicology and Pathology, Research Centre, Grünenthal GmbH, Zieglerstr. 6, W-5100 Aachen, Germany

[CA] Corresponding Author

**AN enteritis, based on a delayed-type hypersensitivity reaction, was induced in TNBS (2,4,4-trinitrobenzenesulphonic acid) sensitized rats by multiple intrajejunal challenge with TNBS via an implanted catheter. This treatment induced chronic inflammation of the distal small intestine characterized by intense hyperaemia, oedema and gut wall thickening as assessed by macroscopic scoring and weighing a defined part of the dissected intestine. Histologically, the inflammatory response included mucosal and submucosal cell infiltration by lymphocytes and histiocytes, transmural granulomatous inflammation with multinucleated cells and activated mesenteric lymph nodes. *Ex vivo* stimulated release of the inflammatory mediator $LTB_4$ in the dissected part of the intestine was increased following TNBS treatment. Drug treatment with sulphasalazine or 5-aminosalicylic acid improved the enteritis score and attenuated TNBS-induced oedema formation and $LTB_4$ production. The applicability and relevance of this new model are discussed with respect to drug development and basic research of inflammatory bowel diseases.**

**Key words:** 5-Amino-salicylic acid, Animal model, Inflammation, Leukotriene $B_4$, Sulphasalazine, TNBS

### Introduction

Inflammatory bowel diseases, Crohn's disease (CD) and ulcerative colitis (UC), represent chronic inflammation of the gastrointestinal tract of unknown aetiology involving immunological events. Recently, these immunological parameters have been described as secondary but may possibly be attributed to the chronicity of the disease.[1–3] Research on the aetiopathogenesis of inflammatory bowel disease and testing of potential therapeutic agents has been hampered by the paucity of reproducible and histopathologically relevant animal models of chronic intestinal inflammation.

Recently, colitis was induced in rats by a single local application of TNBS dissolved in ethanol as a mucosal irritant.[4] This procedure induced a severe and prolonged degenerative inflammation of large parts of the colon. This model shared several aspects of the human inflammatory bowel disease (IBD); and a chronic bowel inflammation, with the typical histopathological infiltration of lymphocytes and histiocytes including granuloma formation and Langhans-type giant cells, was observed. The induction of the disease by a single destructive event is quite different to a more or less continuous or weak stimulation of the immune system by one or more immunogens that might be one of the multiple factors causing human IBDs. We intended to mimic the human disease more closely by realizing the following three-step concept, using TNBS (2,4,4-trinitrobenzenesulphonic acid) as a defined hapten:

1. specific hypersensitivity by active immunization
2. local inflammation by local challenge
3. chronicity by chronic application of the immunogen.

Part of this study has been presented at the '13th European Workshop on Inflammation', May 1991, and will be published as an extended abstract in *Agents and Actions* in 1992.

### Materials and Methods

*Immunization:* Female Sprague–Dawley rats (150–200 g) were sensitized by intradermal injection of 0.8% TNBS (Fluka, Neu-Ulm, Germany), into a shaved area on the back once daily for 3 consecutive days. TNBS was dissolved in 0.05 ml Freund's incomplete adjuvant (FIA; Difco, Detroit, Michigan, USA) together with 1 mg/ml ovalbumin (OA; Sigma, Deisenhofen, Germany). After 18 days the animals received a further intradermal booster injection. Intradermal challenge of 0.08% TNBS in 0.05 ml 0.9% NaCl solution with or without OA, or OA solution without TNBS, was given 14 days

© 1992 Rapid Communications of Oxford Ltd later in order to determine the type and specificity of the immunological reaction.

*Induction of enteritis:* Ten days after the intradermal challenge a flexible polyethylene tube of 0.5 mm diameter was implanted under ketamine anaesthesia (100 mg/kg i.p.) 15 cm proximal to the caecum and emerging at the neck for TNBS or drug administration. After a 10-day recovery phase the animals, kept in separate cages, were treated daily for 3 weeks with 0.08% TNBS in saline (0.2 mg/rat) given intraluminally, control groups receiving only saline. Rats were killed by $CO_2$ asphyxiation 24 h after the last intraluminal application of TNBS. The distal 10 cm of small intestine anterior to the ileocaecocolic junction (5 cm distance to the open end of the catheter) including Peyer's patches were dissected, cut open longitudinally and rinsed with saline (15°C).

*Assessment of enteritis:* Immediately after dissection, the distal small intestine was visually assessed for inflammation by two independent observers according to the enteritis score outlined in Table 1. This scoring system includes the intensity and extent of the redness, or hyperaemia, one of the main parameters of inflammation, where 0 is defined as no visible damage of the whole 10 cm of the distal small intestine, 0.5 = slight redness of less than 50% of the whole 10 cm, 1.0 = slight redness of the whole 10 cm, 1.5 = intense redness of about 25% of the 10 cm, 2.0 = intense redness of about 50% of the whole 10 cm, 2.5 = intense redness of about 75% of the whole 10 cm and 3.0 = intense redness of the whole 10 cm. The dissected gut segments, precisely 10 cm long, were weighed as a parameter of oedema formation and then incubated in 1 ml Tris buffer (50 mM, pH 7.4, 100 mM NaCl, 1 mM $CaCl_2$, 1 mg/ml glucose)/100 mg tissue for 30 minutes at 37°C in a shaking water bath. Following incubation, aliquots of the solutions were centrifuged (13 000 *g*, 2 min, 20°C) and the supernatants stored (−28°C) for determination of leukotriene $B_4$ ($LTB_4$) by radioimmunoassay. Supernatants were used without prior extraction or chromatography and measured by specific radioimmunoassays for $LTB_4$ purchased from Amersham Buchler, Brunswick, Germany.

**Table 1.** Criteria and score for gross morphological damage

| Enteritis score | Gross morphology |
|---|---|
| 0 | No damage |
| 1 | Slight inflammation<br>Slight hyperaemia<br>Visible villi* |
| 2 | Intermediate inflammation<br>Discontinuous hyperaemia<br>Intermediate redness of villi* |
| 3 | Intense inflammation<br>Intense hyperaemia<br>Intense redness of villi,<br>'Tile structure' of villi* |

* Observed under × 15 magnification.

*Histology:* To confirm the visual assessment of inflammation, segments of inflamed and unaffected distal small intestine, obtained from the same animal, were fixed in 10% neutral buffered formalin and processed routinely before embedding in paraffin. The paraffin sections were stained with haematoxylin and eosin and examined by light microscopy.

*Statistical analysis:* Results were expressed as mean ± SEM of (*n*) experiments. Differences between control and inflamed tissue, and influence of drug treatment were compared within each experiment, and statistical significance was calculated by Wilcoxon-Mann-Whitney U-test for unpaired data, two-tailed, the level of significance being taken as $p \leq 0.05$.

*Drug treatment:* Drugs were applied either orally (p.o.) by gavage twice a day, suspended in 1% carboxymethylcellulose (CMC), or intraluminally (i.l.) once a day, suspended in saline. The intervals between oral drug applications were 10–14 h per day, the intervals between intraluminal drug applications were 24 h per day. The intervals between drug and TNBS application were 5 h for oral drug application, and 12 h for intraluminal application. Drugs used were 5-aminosalicylic acid (5-ASA; Heraeus, Karlsruhe, Germany) 21.5 mg/kg/day i.l. or 2 × 21.5 mg/kg/day orally and sulphasalazine (SAZ; Pharmacia) 46.4 mg/kg/day i.l. or 2 × 46.4 mg/kg/day orally. Control groups received only CMC or saline.

### Results

Compared to untreated non-operated animals implantation of a tube did not affect weight gain and well-being of groups of sham-operated animals and groups of animals which additionally received multiple intraluminal application of saline or CMC. In addition, no visible redness or haemorrhage or oedema formation, or any histological difference compared to untreated animals, was detectable except for local granuloma formation just around the fixation points of the tube.

In preliminary experiments a 0.08% solution of TNBS (0.20 mg/rat) was found to cause a reproducible slight inflammation 24 h after a single local administration in sensitized animals, but not in non-sensitized rats. At a lower TNBS concentration (0.01%; 0.025 mg/rat) the inflammation was not reproducible, whereas 0.5% (1.25 mg/rat)

TNBS had a direct inflammatory action on the gut of non-TNBS-sensitized rats.

Investigations on days 7, 14, 21 and 28 of TNBS application were carried out in order to determine the minimum time taken to reach a chronic stage of intestinal inflammation. Only 3 and 4 weeks of application of TNBS resulted in a reproducible state of the inflammatory reaction in each animal. Therefore a 21-day challenge was chosen for the following investigations.

The immunization procedure induced a reproducible and prolonged (9 months) delayed type hyperreactivity against TNBS, and TNBS bound to OA, but not against OA itself. This was examined by repeated monthly intradermal injections of non-irritating doses of TNBS alone or TNBS bound to OA, or OA alone.

Chronic TNBS-induced inflammation of the distal small intestine in rats was characterized by intense hyperaemia and oedema of the mucosal surface 5–15 cm distal to the end of the implanted catheter. Histological examination of this part of the intestine showed inflammatory cell infiltration mainly by lymphocytes and histiocytes in all layers of the intestinal wall. No infiltration of neutrophils was seen in any of the sections obtained, and goblet cells were always intact (Figs 1 and 2). Transmural granulomatous inflammation of the gut wall, with multinucleated cells and activated Peyer's patches, was also observed microscopically (Figs 3 and 4). No differences compared to untreated animals were seen in proximal small intestines or colons from TNBS-treated rats.

Intraluminal TNBS treatment induced moderate to intense inflammation of the distal small intestine as examined according to the scoring system described in Table 1 (see Materials and Methods). Mean values obtained from ten animals per group were 2.35 $\pm$ 0.15 and 2.0 $\pm$ 0.08, after TNBS with orally or intraluminally administered drug-vehicle,

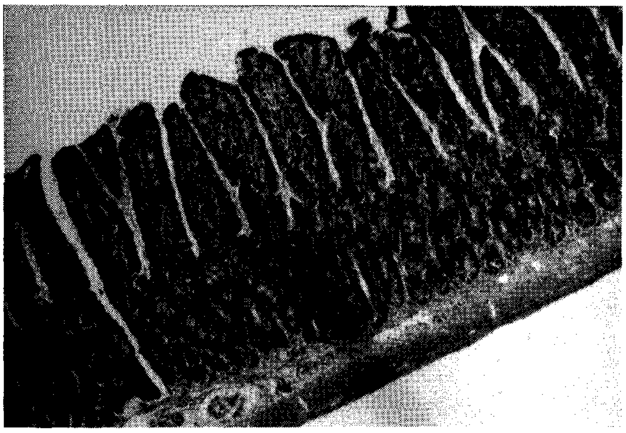

FIG. 1. Histologic section of distal small intestine (ileum) from a control rat. Tissue sample was taken and fixed immediately after sacrifice. Note the intact mucosal cylinder-shaped villi and crypts (epithelial layer). There is also no histologically detectable damage in the submucosa and muscular coat; ×80.

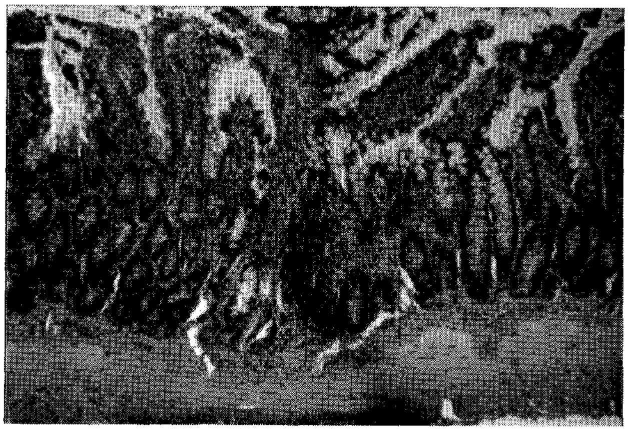

FIG. 2. Paraffin section of ileum after TNBS sensitization and TNBS treatment showing moderate focal mononuclear cell infiltration in the lamina propria and submucosa. Note the tissue disorganization in the area of the inflammatory process. Tissue sample was taken and fixed immediately after sacrifice; ×80.

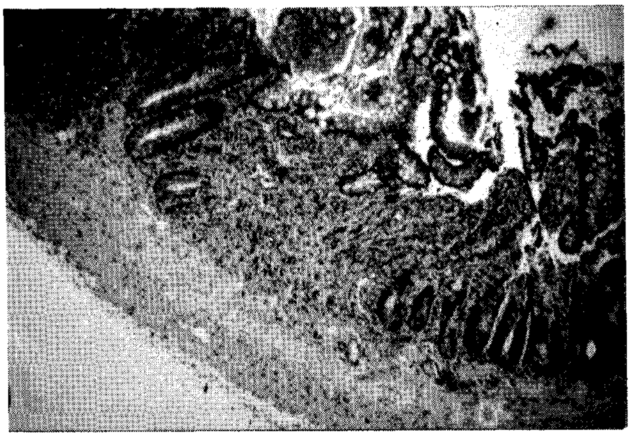

FIG. 3. Section of ileum after TNBS sensitization and TNBS treatment including activated gut-associated lymphoid tissue (Peyer's patch, left upper part of the figure). Note the severe mucosal lymphohistiocytic infiltration and incipient fibroblastic proliferation in the epithelial layer and lamina propria extending through the lamina muscularis mucosae. The area of chronic inflammatory response is lined with intact glandular mucosa. Ileum sample was taken and fixed immediately after sacrifice; ×80.

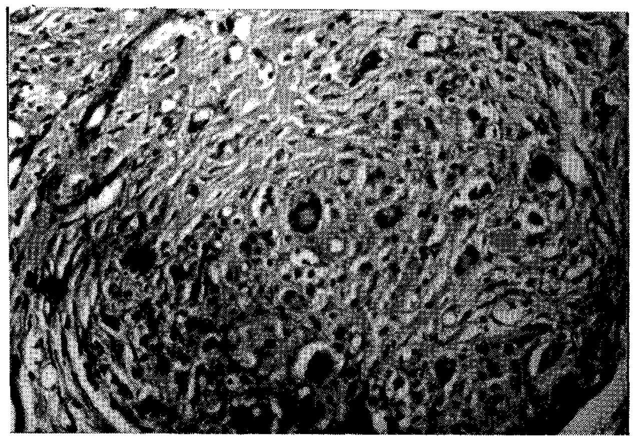

FIG. 4. Granulomatous reaction in the ileum of a TNBS-sensitized and TNBS-treated rat. Ileum sample was taken and fixed immediately after sacrifice. The submucosal granuloma is characterized by compact, sharply outlined aggregates of epithelioid histiocytes and numerous multinucleated Langhans-type giant cells accompanied by lymphocytes of fibroblastic proliferation; ×200.

respectively; and 2.19 $\pm$ 0.16 after TNBS without further drug-vehicle administration (Fig. 5). Ulcerations were not observed in any of the animals treated with 0.08% TNBS.

Local saline instead of TNBS treatment induced no or only slight inflammation in some animals which was also observed in naive animals, see Fig. 5. In non-TNBS-sensitized animals intraluminal TNBS (0.08%) did not induce intense hyperaemia, although some animals showed slight hyperaemia but without any histological signs of inflammation. Chronic TNBS treatment in sensitized animals induced a significant increase in gut weight, which was used as an unspecific parameter of oedema formation or development of granulomatous inflammation. TNBS increased the normal weight of a constant 10 cm of gut from about 730 mg to about 1100 mg (Fig. 6). In sensitized rats chronic TNBS treatment also significantly increased $LTB_4$ production of incubated gut sections in comparison to saline-treated groups, 255 $\pm$ 51.8 pg/g tissue (TNBS) vs 14 $\pm$ 2.6 pg/g tissue (NaCl) (Fig. 7).

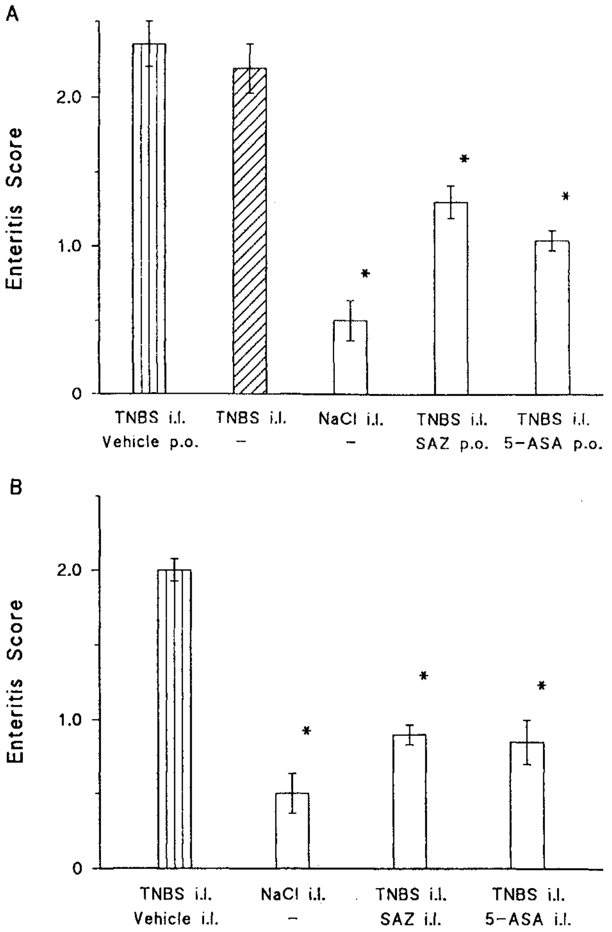

FIG. 5. Effect of (A) orally (p.o.) and (B) intraluminally (i.l.) supplied sulphasalazine (SAZ) and 5-aminosalicylic acid (5-ASA) on the enteritis score in TNBS-induced inflammation of distal small intestine. SAZ was used in doses of 2 x 46.4 mg/kg/day p.o. (A) and 46.4 mg/kg/day i.l. (B). 5-ASA was used in doses of 2 x 21.5 mg/kg/day p.o. (A) and 21.5 mg/kg/day i.l. (B). Each bar represents the mean ($\pm$SEM) of ten rats/group. * $p \leq 0.05$ *vs.* vehicle-treated TNBS-sensitized animals.

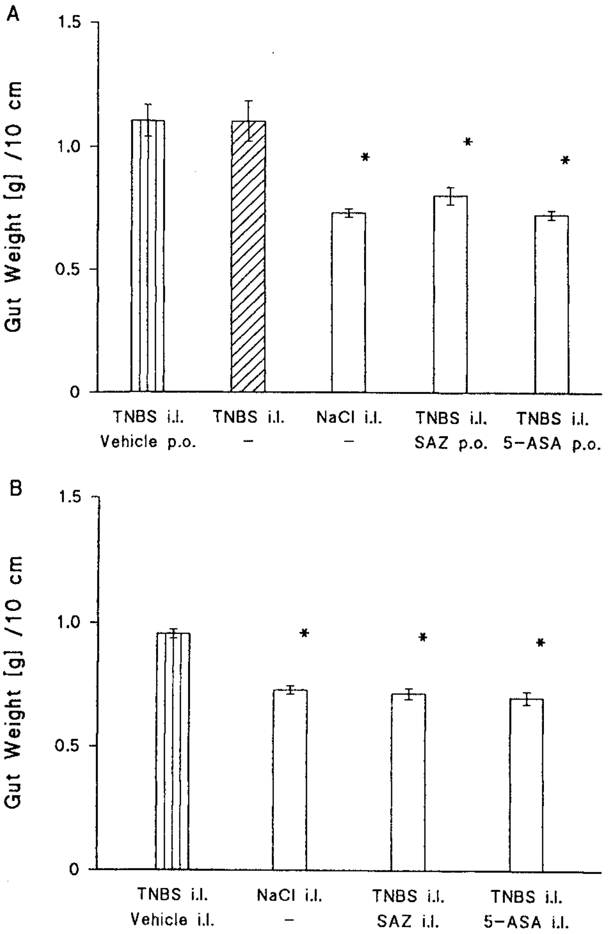

FIG. 6. Effect of (A) orally (p.o.) and (B) intraluminally (i.l.) applied sulphasalazine (SAZ) and 5-aminosalicylic acid (5-ASA) on gut weight in TNBS-induced inflammation of distal small intestine. SAZ was used in doses of 2 x 46.4 mg/kg/day p.o. (A) and 46.4 mg/kg/day i.l. (B). 5-ASA was used in doses of 2 x 21.5 mg/kg/day p.o. (A) and 21.5 mg/kg/day i.l. (B). Each bar represents the mean ($\pm$SEM) of ten rats/group. * $p \leq 0.05$ *vs.* vehicle-treated TNBS-sensitized animals.

Oral or intraluminal treatment with sulphasalazine (SAZ) and 5-aminosalicylic acid (5-ASA), administered in doses of 2 $\times$ 46.4 or 46.4 mg/kg/day (i.l.), or 2 $\times$ 21.5 mg/kg/day (p.o.) or 21.5 mg/kg/day (i.l.), improved all parameters used in this model. Enteritis score was decreased from 2.19 to 1.3 or 1.04 by oral treatment, and from 2.0 to 0.9 or 0.85 by intraluminal treatment with SAZ or 5-ASA (Fig. 5). Intraluminal administration of half the oral dose seemed to induce an equipotent anti-inflammatory effect on the enteritis score. The TNBS-induced increase in gut weight was reduced by oral and intraluminal treatment with SAZ and 5-ASA to such an extent that there was no detectable difference between these and sham-operated, saline-treated, TNBS-sensitized animals (Fig. 6). $LTB_4$ production by incubated gut sections from treated animals was reduced more markedly by oral than by intraluminal SAZ or 5-ASA treatment (Fig. 7A). Oral treatment (Fig. 7) induced a 90% or 85% reduction as compared to TNBS control values, whereas intraluminal treatment only

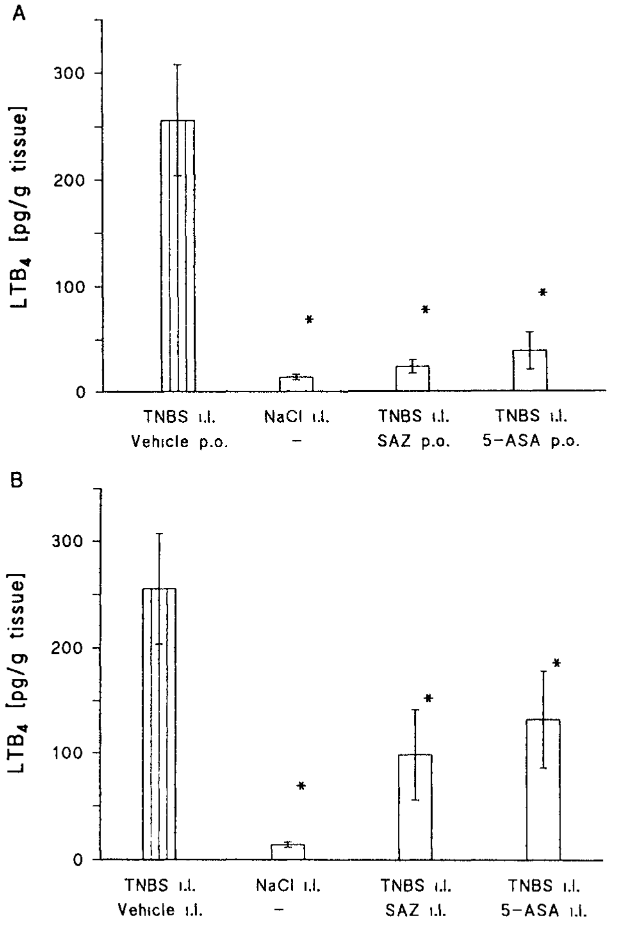

FIG. 7. Effect of (A) orally (p.o.) and (B) intraluminally (i.l.) applied sulphasalazine(SAZ) and 5-aminosalicylic acid (5-ASA) on *ex vivo* leukotriene $B_4$ production of TNBS-treated distal small intestine. SAZ was used in doses of 2 × 46.4 mg/kg/day p.o. (A) and 46.4 mg/kg/day i.l. (B). 5-ASA was used in doses of 2 × 21.5 mg/kg/day p.o. (A) and 21.5 mg/kg/day i.l. (B). Each bar represents the mean (±SEM) of ten rats/group. * $p \leq 0.05$ *vs.* vehicle-treated TNBS-sensitized animals.

resulted in a 60% or 50% reduction (Fig. 7B). Histological examination of drug-treated groups showed no or only few and small inflamed segments in all animals tested.

### Discussion

Since IBD-like diseases do not occur naturally in animals, different types of exogenous irritants were used to induce inflammatory reactions of the gut. Though the importance of the immune system in the course of human IBDs is still unknown, an immunological involvement cannot be excluded.[1–3,5–8] Therefore, an immunological type of irritant seems to be a suitable tool for inducing an IBD-like intestinal inflammation in rats.

DNCB and TNCB (di- or tri-nitrochlorobenzene) are very potent haptens. When bound to tissue protein they have been shown to elicit cell-mediated immune responses.[9–15] Animals can be sensitized by an application of DNCB or TNCB to their skin, which induces a reproducible and prolonged delayed type of hypersensitivity. In contrast to DNCB and TNCB, TNBS, the hapten used in this investigation, is water-soluble;[15] this property is required for local administration by a catheter. TNBS by itself did not induce any immunological reaction, as mentioned in the results. However, when TNBS was bound to OA as a carrier and reinforced by Freund's adjuvant, an immunological response similar to that after DNCB or TNCB was obtained. The specificity of the TNBS-induced sensitization was investigated by skin injections of low and inactive doses of the hapten, with or without binding to OA, or OA alone. A clear delayed type of hypersensitivity was observed only against the hapten TNBS. It was reproducible for more than 9 months as examined by monthly skin tests.

Local treatment of the intestine with TNBS following immunization was shown to induce an inflammation of the rat gut very similar to human Crohn's disease. In particular, the histologically observed infiltration of lymphocytes and histiocytes is exactly as described for the human disease.[18] In addition, the induced transmural inflammatory reaction involving all layers of the intestinal wall, including the mesenteric lymph nodes, the occurrence of granulomas with multinucleated giant cells, and the fact that all goblet cells remain intact[18] are features of the animal model that show similarity to Crohn's disease.

The macroscopic description of the inflammatory reaction of the distal small intestine treated by multiple local TNBS administration includes intensity and redness or haemorrhage as one of the main parameters of inflammation as outlined in the methods. This scoring system was chosen as it is easy to determine, robust and is only minimally subject to observer bias. Ulceration was never seen after low dose TNBS treatment but can be induced by higher doses (personal observation).

In animals not previously sensitized to TNBS no transmural chronic inflammation could be induced by intrajejunal administration of TNBS, although TNBS was used in concentrations up to 0.5%. These results support the hypothesis of immune involvement in the aetiology of inflammatory bowel diseases,[1,5,9] in which conversion of lymphocyte reactivity is discussed as a possible pathogenic parameter.

One main difference from Crohn's disease is the non-occurrence of a discontinuous inflammation characterized by the typically observed pattern of remission and relapse, and the typical mixture of chronic and acute inflammation. However, the prolonged reactivity against TNBS in the present investigation may provide a chance to mimic the course of the human disease by discontinuous intraluminal application of the hapten in sensitized animals and variation of the concentration of the hapten.

The efficacy of 5-ASA and sulphasalazine in this model is another aspect that emphasizes the similarity of the model to the human disease. Both drugs represent the most important clinically used drugs for IBD, and in this investigation they were used at a dose which is effective in human treatments. The mentioned differences between i.l. and p.o. administration on $LTB_4$ and enteritis score were difficult to rationalize. Reduction of $LTB_4$ was more pronounced by oral than intraluminal drug administration, whereas reduction of the enteritis score was more pronounced by intraluminal drug administration. The reason for these differences is not yet clear. Future investigations and dose-dependent studies are needed, especially as the overall efficacy of sulphasalazine in this model is higher than expected considering that only colonic bacteria can split the molecule into sulphapyridine and the active 5-ASA moiety.

The inflammation mediator $LTB_4$,[19–21] often found to be increased in patients with inflammatory bowel diseases,[22,23] was additionally determined in this model. Levels of $LTB_4$ in TNBS-treated animals were significantly increased as compared to controls. Another characteristic of the human disease is therefore demonstrated in this animal model. Again, the relevance of this parameter for the aetiology or the course of the disease remains unclear as it is not known whether it is a primary or secondary event in the process of the disease. In addition, the determination of lipoxygenase product formation of the intestine should be tested by direct extraction of the inflamed tissue especially by HPLC-techniques in order to avoid artificial errors. Due to the multifactorial origin of the human disease the determination of other mediators and cytokines in this model would be of great interest and helpful for further characterization of the model. From the histological findings the increase of $LTB_4$-like material determined in this model did not seem to be caused by neutrophils; only macrophages or mast cells will be assumed as the sources for leukotriene production.

In contrast to other immunologically-induced chronic inflammations of the gut as animal models of IBD,[4,9–14] chronicity of the inflammatory reaction in this model was induced by multiple instead of one[4] or few[10,13,14] local administrations of the immunogen. Another difference is a 3 week instead of 3 day[10,13,14] time schedule for the local challenge procedure. This proposed continuous and weak stimulation of the immune system by one specific immunogen seems to be an opportunity to mimic the human disease more closely and the model has more features in common with human IBDs than non-immunological and acute models.[24]

In conclusion, the presented animal model of IBD is rather complex and time-consuming due to the immunization procedure and the sustained local treatment. However, this challenge relates to the characteristics of a chronic disease and provides a reproducible model of chronic and immunologically-induced inflammatory bowel disease.

### References

1. von Herbay A, Gebbers JO, Otto HF. Immunopathology of Ulcerative Colitis: A review. *Hepato-Gastroenterol* 1990; **37:** 99–107.
2. Aparicio-Pages MN, Verspaget HW, Pena AS, Weterman IT, de Bruin PA, Mierement-Ooms MA, van der Zon JM, van Tol EA, Lamers CB. In Vitro Cellular Cytotoxicity in Crohn's Disease and Ulcerative Colitis: Relation with disease activity and treatment, and the effect of recombinant gamma-interferon. *J Clin Lab Immunol* 1989; **29:** 119–124.
3. Fiocchi C. Immune Events Associated with Inflammatory Bowel Disease. *Scand J Gastroenterol* 1990; **25 (Suppl. 172):** 4–12.
4. Morris GP, Beck PL, Herridge MS, Depew WT, Szewczuk MR, Wallace JL. Hapten-Induced Model of Chronic Inflammation and Ulceration in the Rat Colon. *Gastroenterology* 1989; **96:** 795–803.
5. Raedler A, Schreiber S. Immunology of Ulcerative Colitis. *Hepato-Gastroenterol* 1989; **36:** 213–218.
6. Beeken WL, Sessions JT, Bozymski EM. Correlations between Clinical, Blood Leukocyte, and Skin Test Data in the National Cooperative Crohn's Disease Study. *Gastroenterology* 1979; **77:** 921–924.
7. Beeken W, Andre-Ukena SS, Gundel RM. Histochemical and Cytotoxicity Studies of Peripheral Blood Monocytes in Crohn's Disease *Gastroenterology* 1980; **78:** 1138–1139.
8. Chambers TJ, Morson BC. The Granuloma in Crohn's Disease. *Gut* 1979; **20:** 269–274.
9. Boughton-Smith NK, Whittle BJR. Increased Metabolism of Arachidonic Acid in an Immune Model of Colitis in Guinea-Pigs. *Br J Pharmacol* 1985; **86:** 439–446.
10. Bicks RO, Rosenberg EW. A Chronic Delayed Hypersensitivity Reaction in the Guinea-Pig Colon. *Gastroenterology* 1964; **46:** 543–549.
11. Bicks RO, Brown G, Hickey D, Rosenberg EW. Further Observations on a Delayed Hypersensitivity Reaction in the Guinea-Pig Colon. *Gastroenterology* 1965; **48:** 425–429.
12. Bicks RO, Azar MM, Rosenberg EW, Dunham WG, Luther JS. Delayed Hypersensitivity Reactions in the Intestinal Tract. I. Studies of 2,4,Dinitrochlorobenzene-caused Guinea-Pig and Swine Colon Lesion. *Gastroenterology* 1967; **53:** 422–436.
13. Rabin BS, Rogers SJ. A Cell-Mediated Immune Model of Inflammatory Bowel Disease in the Rabbit. *Gastroenterology* 1978; **75:** 29–33.
14. Bruce SR. Animal Model of Human Disease: Immunologic model of inflammatory bowel disease. *Am J Pathol* 1980; **99:** 253–256.
15. Stein-Streilein J, Lipscomb MF, Fisch H, Whitney PL. Pulmonary Interstitial Fibrosis Induced in Hapten-Immune Hamsters. *Am Rev Respir Dis* 1987; **136:** 119–123.
16. Kusugami K, Youngman KR, West GA, Fiocchi C. Intestinal Immune Reactivity to Interleukin 2 differs among Crohn's Disease, Ulcerative Colitis, and Controls. *Gastroenterology* 1989; **97:**1–9.
17. Kramer JK, Depew WT, Szewczuk MR. T-Cell Immunoregulation in Patients with Inflammatory Bowel Disease. II. Enhanced Suppressor T-Cell Activity in Ulcerative Colitis. *J Clin Lab Immunol* 1988; **25:** 19–27.
18. Rabbins JL, Cotran RS. *Pathologic Basis of Disease*, Philadelphia. W.B. Saunders Co., 1979; 958–987.
19. Ford-Hutchinson AW. Leukotriene Involvement in Pathological Processes *J Allerg Clin Pharm* 1984; **74:** 437–445.
20. Samuelsson B. The Leukotrienes. *Pure Appl Chem* 1981; **53:** 1203–1208.
21. Parker CW. Various Roles of Lipids and Lipid Metabolizing Enzymes in Inflammatory Processes and their Possible Implications for Therapy. *Am Rev Respir Dis* 1987; **135:** S22–S25.
22. Stenson WF. Role of Eicosanoids as Mediators of Inflammation in Inflammatory Bowel Disease. *Scand J Gastroenterol* 1990; **25:** 13–18.
23. Fretland DJ, Djuric SW, Gaginella TS. Eicosanoids and Inflammatory Bowel Disease; Regulation and Prospects for Therapy. *Prostagland Leuk Essent Fatty Acids* 1990; **41:** 215–235.
24. Boxenbaum HG, Dairman W. Evaluation of an Animal Model for the Screening of Compounds Potentially Useful in Human Ulcerative Colitis: Effect of Salicylazosulfapyridine and Prednisolone on Carrageenan-induced Ulceration of the Large Intestine of the Guinea-Pig. *Drug Dev Ind Pharm* 1977; **3:** 121–130.

ACKNOWLEDGEMENTS. The excellent technical assistance of Mrs E. Lerch, Ms E. Haase and Mrs A. Würfler and expecially of Mr G. Haase gratefully acknowledged.

**Received 15 January 1992; accepted in revised form 31 January 1992**
